# Unilateral uveitis, cataract and retinal detachment following low-voltage electrical injury

**DOI:** 10.1186/s41038-015-0020-x

**Published:** 2015-10-15

**Authors:** Rahmi Duman, Sadık Görkem Çevik, Ayşe Tüfekçi

**Affiliations:** 1 Department of Ophthalmology, Sevket Yılmaz Training and Research Hospital, Bursa, Turkey; 2 Çamlıtepe Mah, Kıbrıs Caddesi 9/4, Çankaya, Ankara Turkey

## Abstract

A 39-year-old woman presented with a gradual worsening of vision in the right eye 1 month after a low-voltage household electrical injury. A slit-lamp examination showed non-granulomatous anterior uveitis with nuclear cataract and an ultrasound examination also showed total retinal detachment. In this letter, we present a rare complication of electrical injury demonstrated as unilateral uveitis, cataract and retinal detachment in a 39-year-old woman.

## Findings

The paper presents a rare complication of electrical injury demonstrated as unilateral uveitis, cataract and retinal detachment in a 39-year-old woman. Electrical injuries are relatively uncommon. Electrical accidents can be classified according to whether the current is high or low. Low-voltage electrical injuries are cases of exposure of less than 1000 V and usually happen at home. Electrical injuries can cause a wide variety of complications depending on the voltage, current, pathway and duration of contact. Damages to the eye, occurring due to electric shock, rarely happen as a result of accidents affecting the head. Herein, we report a patient with a unilateral uveitis, cataract and retinal detachment, developed during the early period pursuant to a low-voltage electrical injury. Written informed consent was obtained from the patient for publication of this paper and any accompanying images.

**Fig. 1:**
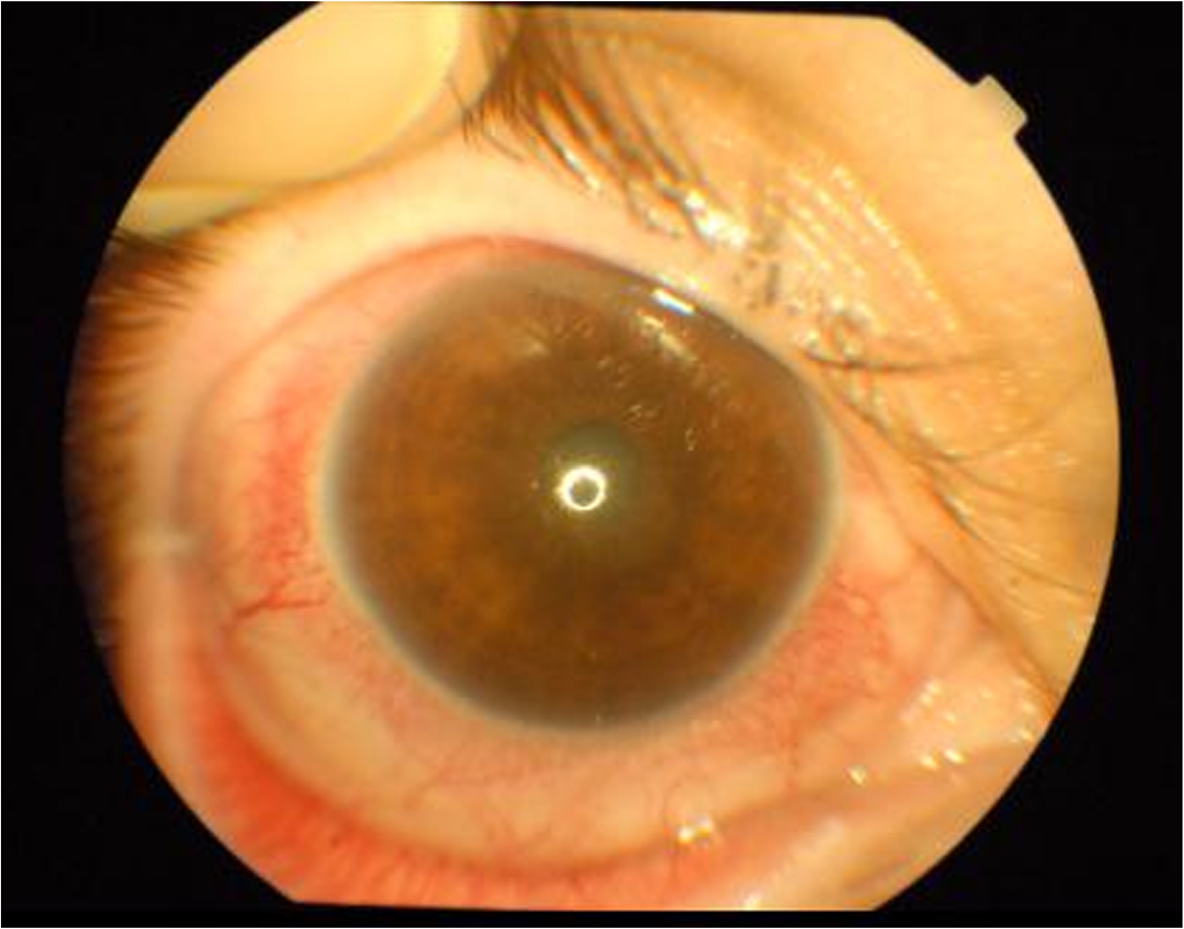
Photograph showed non-granulomatous anterior uveitis with nuclear cataract and ciliary injection in the right eye

**Fig. 2:**
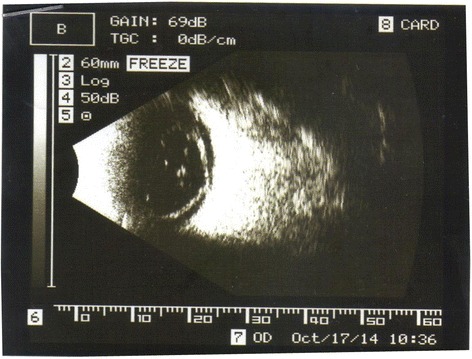
An ultrasound examination showed total retinal detachment

A 39-year-old woman was brought to the emergency center at Şevket Yılmaz Training and Research Hospital as she had a low-voltage household electrical injury. When the patient was brought to the emergency room, she had developed a cardiac arrest. She was intubated and placed under treatment in the intensive care unit. She lost consciousness for 1 week following the accident. At the time of admission, she had third-degree burns on the right side of her leg. An ocular examination given in the course of admission revealed normal findings without any corneal or lenticular opacity and evidence of penetration or perforation of the globes. Apart from those findings, no symptoms of electrical burns were observed on either of the eyelids and around the eyes. During the four weeks of hospitalization, the patient suffered from a gradual worsening of vision in the right eye. Her vision in right eye was limited to perception of hand motions, with an intraocular pressure of 13 mmHg in each eye. In her right eye, a slit-lamp examination showed non-granulomatous anterior uveitis with nuclear cataract (Fig. 1). Fundus examination could not be carried out on the right eye. An ultrasound examination also showed total retinal detachment (Fig. 2). The patient reported normal visual acuity in each eye before the injury. There were no pathological findings in the left eye. There were no systemic or methabolic changes which may cause cataract. The patient was treated with topical dexametazon and %1 siklopentolat HCL ophthalmic solution four times daily and she was referred to another center for vitreoretinal and cataract surgery. After cataract surgery combined to pars plana vitrectomy with gas tamponade the patient’s postoperative Snellen visual acuity at first month visit was 0.2.

The pathophysiologic features of electrically-induced ocular injury are complex and the amount of tissue destruction depends on several variables, the duration of electric current passage, the orientation of the cells in the current path, their location, and other factors including the voltage, amperage and resistance [1]. Electrically-induced ocular injury has been associated with many pathologic changes such as cataract, macular edema, eyelid edema, corneaepitelial keratitis, chemosis and pupillary abnormalities [2–5]. Among these, cataract is the most common complication [5–7]. Less damage occurs in low resistant parts of the eye like retina and optic nerve [8]. The lens is the most sensitive tissue to electrical current and the resultant induced heat in the eye [9].

In the previous studies of electrically-induced ocular injury the rates of cataract have been documented to range from 1 % to 6 % [5, 10, 11]. Ferreiro et al. reported that the voltage does not have any influence on the severity of the cataract and the current pathway, as well as its points of entry, does not show any relation with the presence of renal failure, cardiac arrhythmia and cataracts [10]. İn other study Solem et al. reported that the patients who had the cephalic region had higher probability of developing cataracts [11]. Boozalis et al. reported that eight patients with cataracts and determine the characteristic changes in lenses. All four patients with cataractous changes had characteristic anterior subcapsular opacifications, except for one patient who presented with a dense white opacified lens [5]. İn our patient we detect ipsilateral nuclear cataract with relation between its presence and the involvement of the ipsilateral region.

Uveitis, cataract and retinal detachment were detected in our patient during the four weeks of hospitalization. Previous studies have generally reported cataract formation as a late complication [5–8]. Unilateral cataract may rarely be observed during the early recovery period of a high-voltage electrical injury, and there are a few reports with unilateral ocular complications [1, 12, 13]. The cataract appeared earlier and progressed faster in the eye nearer the site of the electric shock [12, 13].

Several mechanisms have been postulated to cause retinal detachment, including mechanical, thermal injury, or inflammation [9, 14]. The exact mechanism of unilateral uveitis, cataract and retinal detachment formation after electrical injury is not known. Electrical current might have transmitted only to the right eye or a sudden mechanical injury of vitreous may have resulted tractional retinal detachment in our patient . Also, heat generated by the passage of a current through the eye may cause various cellular or intercullular changes which possibly result in uveitis, cataract and retinal detachment [10, 15].

In the cases of electrical injuries, physicians should be alert to these rare complications. Patients who have experienced electrical injuries that affect especially the head and the neck should be monitored regularly by an ophthalmologist in both early and late period.
